# Dr. James H. Sternberg

**Published:** 1912-05

**Authors:** 


Dr. James H. Sternberg of Waterloo, was found dead in bed, March 26, 1912, aged 78. He was a graduate of Jefferson, 1856.

